# 7-Nitro-2-phenyl­imidazo[2,1-*b*][1,3]benzo­thia­zole

**DOI:** 10.1107/S1600536814000476

**Published:** 2014-01-15

**Authors:** Alexander S. Bunev, Elena V. Sukhonosova, Vladimir E. Statsyuk, Gennady I. Ostapenko, Victor N. Khrustalev

**Affiliations:** a Department of Chemistry and Chemical Technology, Togliatti State University, 14 Belorusskaya St, Togliatti 445667, Russian Federation; b Department of Organic, Bioorganic and Medicinal Chemistry, Samara State University, 1 Akademician Pavlov St, Samara 443011, Russian Federation; c X-Ray Structural Centre, A.N. Nesmeyanov Institute of Organoelement Compounds, Russian Academy of Sciences, 28 Vavilov St, B-334, Moscow 119991, Russian Federation

## Abstract

In the title mol­ecule, C_15_H_9_N_3_O_2_S, the central imidazo[2,1-*b*][1,3]benzo­thia­zole heterotricyclic unit is essentially planar (r.m.s. deviation = 0.021 Å). The terminal phenyl ring and nitro group are twisted by 9.06 (1) and 11.02 (4)°, respectively, from the mean plane of the heterotricycle. In the crystal, mol­ecules are linked by π–π stacking inter­actions into columns along [100]; the inter­planar distance between neighboring imidazo[2,1-*b*][1,3]benzo­thia­zole planes within the columns is 3.370 (2) Å. Furthermore, the columns interact with each other by secondary S⋯O [2.9922 (10) and 3.1988 (11) Å] inter­actions, forming a three-dimensional framework.

## Related literature   

For applications of imidazo[2,1–*b*][1,3]benzo­thia­zoles, see: Ager *et al.* (1988 ▶); Sanfilippo *et al.* (1988 ▶); Barchéchath *et al.* (2005 ▶); Andreani *et al.* (2008 ▶); Chao *et al.* (2009 ▶); Kumbhare *et al.* (2011 ▶); Chandak *et al.* (2013 ▶). For the crystal structures of related compounds, see: Landreau *et al.* (2002 ▶); Adib *et al.* (2008 ▶); Fun, Asik *et al.* (2011 ▶); Fun, Hemamalini *et al.* (2011 ▶); Ghabbour *et al.* (2012 ▶); Bunev *et al.* (2013 ▶).
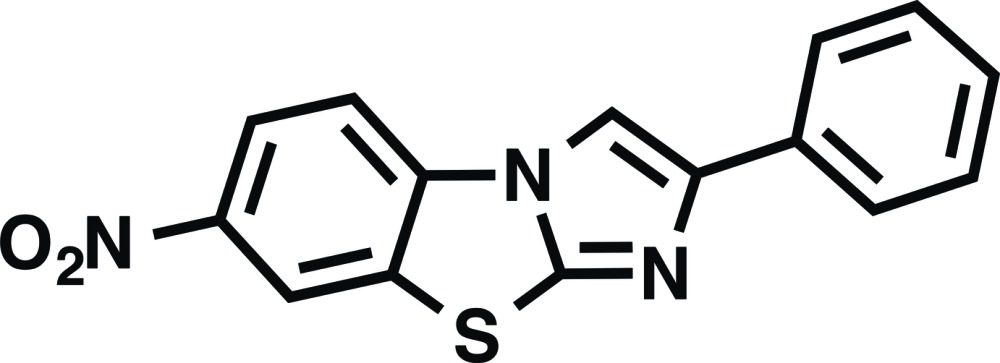


## Experimental   

### 

#### Crystal data  

C_15_H_9_N_3_O_2_S
*M* *_r_* = 295.32
Monoclinic, 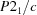

*a* = 6.8068 (3) Å
*b* = 21.0244 (9) Å
*c* = 9.0699 (4) Å
β = 105.077 (1)°
*V* = 1253.30 (9) Å^3^
*Z* = 4
Mo *K*α radiation
μ = 0.27 mm^−1^
*T* = 120 K
0.30 × 0.10 × 0.10 mm

#### Data collection  

Bruker APEXII CCD diffractometer
Absorption correction: multi-scan (*SADABS*; Bruker, 2003 ▶) *T*_min_ = 0.924, *T*_max_ = 0.974
19380 measured reflections
4586 independent reflections
3740 reflections with *I* > 2σ(*I*)
*R*_int_ = 0.041

#### Refinement  

*R*[*F* ^2^ > 2σ(*F* ^2^)] = 0.041
*wR*(*F* ^2^) = 0.110
*S* = 1.03
4586 reflections
190 parameters
H-atom parameters constrained
Δρ_max_ = 0.54 e Å^−3^
Δρ_min_ = −0.38 e Å^−3^

### 

Data collection: *APEX2* (Bruker, 2005 ▶); cell refinement: *SAINT* (Bruker, 2001 ▶); data reduction: *SAINT*; program(s) used to solve structure: *SHELXTL* (Sheldrick, 2008 ▶); program(s) used to refine structure: *SHELXTL*; molecular graphics: *SHELXTL*; software used to prepare material for publication: *SHELXTL*.

## Supplementary Material

Crystal structure: contains datablock(s) global, I. DOI: 10.1107/S1600536814000476/rk2421sup1.cif

Structure factors: contains datablock(s) I. DOI: 10.1107/S1600536814000476/rk2421Isup2.hkl

Supporting information file. DOI: 10.1107/S1600536814000476/rk2421Isup3.cml

CCDC reference:

Additional supporting information:  crystallographic information; 3D view; checkCIF report

## Figures and Tables

**Fig. 1.**
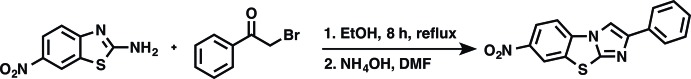
The synthesis of 7-nitro-2-phenylimidazo[2,1-b][1,3]benzothiazole.

**Fig. 2.**
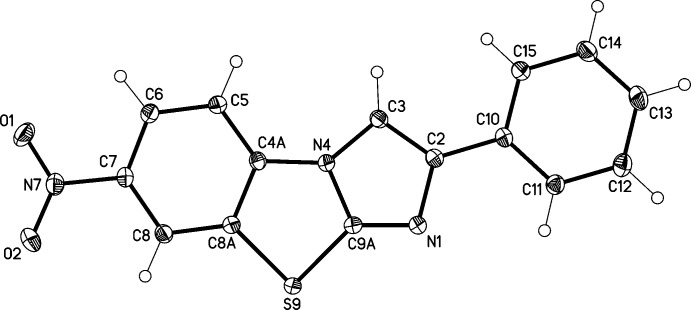
Molecular structure with the atom numbering scheme of I. Displacement ellipsoids are presented at the 50% probability level. H atoms are depicted as a small spheres of arbitrary radius.

**Fig. 3.**
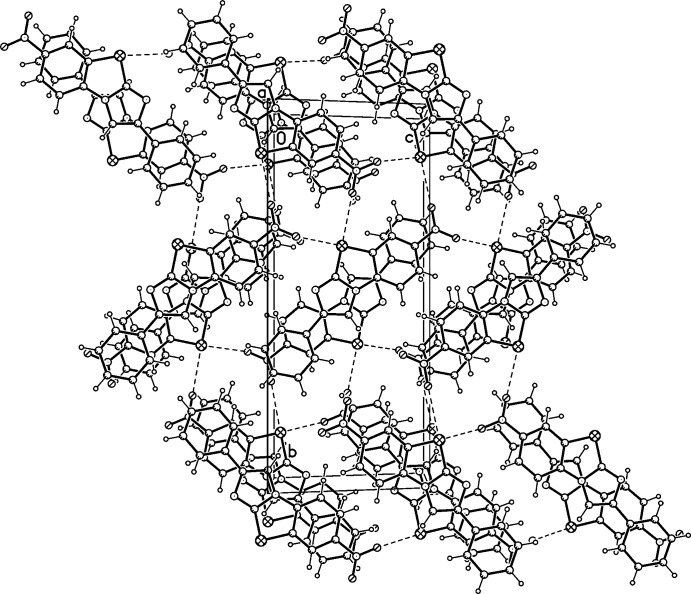
The crystal packing of I demonstrating the columns along the a axis. Dashed lines indicate the intermolecular secondary S···O interactions.

## supplementary crystallographic information

### 1. Comment

Imidazo[2,1–*b*][1,3]benzothiazole are of great interest due to their biological properties. These compounds and their derivatives demonstrate the antitumor (Andreani *et al.*, 2008), antiallergic (Ager *et al.*, 1988), anesthetic (Sanfilippo *et al.*, 1988) and anticancer (Kumbhare *et al.*, 2011) activities as well as the inhibition activity of apoptosis in testiculargerm cells (Chandak *et al.*, 2013), lymphocytes (Barchéchath *et al.*, 2005), and FMS–like tyrosine kinase–3 (FLT3) (Chao *et al.*, 2009).

In this work, a 7–nitro–2–phenylimidazo[2,1–*b*][1,3]benzothiazole, C_15_H_9_N_3_O_2_S, (I) was prepared by the reaction of 2–amino–6–nitro–1,3–benzothiazole with 2–bromo–1–phenylethanone (Fig. 1), and its structure was unambiguously established by the *X*–ray diffraction study (Fig. 2).

The bond lengths and angles within the molecule of I are in a good agreement with those found in the related compounds (Landreau *et al.*, 2002; Adib *et al.*, 2008; Fun, Asik *et al.*, 2011; Fun, Hemamalini *et al.*, 2011; Ghabbour *et al.*, 2012; Bunev *et al.*, 2013). The central imidazo[2,1–*b*][1,3]benzothiazole tricycle in I is essentially planar (r.m.s. deviation is 0.021Å). The terminal phenyl ring and nitro–group are twisted at 9.06 (1) and 11.02 (4)°, respectively, from the mean plane of the tricycle.

In the crystal, the molecules of I are linked by the intermolecular π···π–stacking interactions into columns along [100] (Fig. 3). The molecules within the columns are arranged alternatively by their planar rotation of 180° (Fig. 3). The interplane distance between neighboring imidazo[2,1–*b*][1,3]benzothiazole planes is 3.370 (2)Å). Further the columns are bound to each other by the intermolecular secondary S9···O1^i^ (3.1988 (11)Å) and S9···O2^ii^ (2.9922 (10)Å) interactions into three–dimensional framework (Fig. 3). Symmetry codes: (i) *x*, *y*, 1+*z*; (ii) *x*, 1.5-*y*, 0.5-*z*.

### 2. Experimental

The mixture of 6–nitrobenzothiazol–2–amine (1.95 g, 10 mmol) and 2–bromo–1–phenylethanone (1.99 g, 10 mmol) was dissolved in ethanole (40 ml). The reaction mixture was heated under reflux for 8 h. The resulting precipitate was collected after cooling to room temperature and dissolved in *DMF* (20 ml). The warm solution basified with 20% NH_4_OH (20 ml) yielded the expected imidazo[2,1–*b*][1,3]benzothiazole I after cooling at room temperature. The basified solution was extracted with CH_2_Cl_2_ (3×50 ml), the organic phase was dried (Na_2_SO_4_) and evaporated in *vacuo*. The residue crystallized from *DMF*. Yield is 82%. The single–crystal of the product I was obtained by slow crystallization from *DMF*. M.p. = 539–541 K. IR (KBr), ν/cm^-1^: 3131, 3073, 1580, 1523, 1501, 1337, 1144, 815, 714. ^1^H NMR (500 MHz, DMSO–*d_6_*, 304 K): 7.36–7.29 (m, 3H, CH), 7.95 (d, 1H, *J* = 8.9, CH), 8.18 (c, 1H, CH), 8.30 (d, 1H, *J* = 8.9, CH), 8.54 (dd, 2H, *J* = 2.2, CH). Anal. Calcd for C_15_H_9_N_3_O_2_S: C, 61.01; H, 3.07. Found: C, 61.10; H, 3.12.

### 3. Refinement

All hydrogen atoms were placed in the calculated positions with C—H = 0.95Å and refined in the riding model with fixed isotropic displacement parameters: *U*_iso_(H) = 1.2*U*_eq_(C)].

### Crystal data

**Table:**

C_15_H_9_N_3_O_2_S	*F*(000) = 608
*M**_r_* = 295.32	*D*_x_ = 1.565 Mg m^−^^3^
Monoclinic, *P*2_1_/*c*	Melting point = 539–541 K
Hall symbol: -P 2ybc	Mo *K*α radiation, λ = 0.71073 Å
*a* = 6.8068 (3) Å	Cell parameters from 6622 reflections
*b* = 21.0244 (9) Å	θ = 2.5–32.7°
*c* = 9.0699 (4) Å	µ = 0.27 mm^−^^1^
β = 105.077 (1)°	*T* = 120 K
*V* = 1253.30 (9) Å^3^	Prism, yellow
*Z* = 4	0.30 × 0.10 × 0.10 mm

### Data collection

**Table:**

Bruker APEXII CCD diffractometer	4586 independent reflections
Radiation source: fine–focus sealed tube	3740 reflections with *I* > 2σ(*I*)
Graphite monochromator	*R*_int_ = 0.041
φ and ω scans	θ_max_ = 32.7°, θ_min_ = 1.9°
Absorption correction: multi-scan (*SADABS*; Bruker, 2003)	*h* = −10→10
*T*_min_ = 0.924, *T*_max_ = 0.974	*k* = −31→30
19380 measured reflections	*l* = −13→13

### Refinement

**Table:**

Refinement on *F*^2^	Primary atom site location: structure-invariant direct methods
Least-squares matrix: full	Secondary atom site location: difference Fourier map
*R*[*F*^2^ > 2σ(*F*^2^)] = 0.041	Hydrogen site location: inferred from neighbouring sites
*wR*(*F*^2^) = 0.110	H-atom parameters constrained
*S* = 1.03	*w* = 1/[σ^2^(*F*_o_^2^) + (0.058*P*)^2^ + 0.471*P*] where *P* = (*F*_o_^2^ + 2*F*_c_^2^)/3
4586 reflections	(Δ/σ)_max_ = 0.001
190 parameters	Δρ_max_ = 0.54 e Å^−^^3^
0 restraints	Δρ_min_ = −0.38 e Å^−^^3^

### Special details

**Table:**

Geometry. All s.u.'s (except the s.u. in the dihedral angle between two l.s. planes) are estimated using the full covariance matrix. The cell s.u.'s are taken into account individually in the estimation of s.u.'s in distances, angles and torsion angles; correlations between s.u.'s in cell parameters are only used when they are defined by crystal symmetry. An approximate (isotropic) treatment of cell s.u.'s is used for estimating s.u.'s involving l.s. planes.
Refinement. Refinement of *F*^2^ against ALL reflections. The weighted *R*–factor *wR* and goodness of fit *S* are based on *F*^2^, conventional *R*–factors *R* are based on *F*, with *F* set to zero for negative *F*^2^. The threshold expression of *F*^2^ > σ(*F*^2^) is used only for calculating *R*–factors(gt) *etc*. and is not relevant to the choice of reflections for refinement. *R*–factors based on *F*^2^ are statistically about twice as large as those based on *F*, and *R*–factors based on ALL data will be even larger.

### Fractional atomic coordinates and isotropic or equivalent isotropic displacement parameters (Å^2^)

**Table:**

	*x*	*y*	*z*	*U*_iso_*/*U*_eq_	
N1	0.28591 (16)	0.51605 (5)	0.71585 (12)	0.01553 (19)	
C2	0.28524 (17)	0.45307 (5)	0.66432 (13)	0.0135 (2)	
C3	0.25724 (17)	0.45089 (5)	0.50820 (14)	0.0142 (2)	
H3	0.2503	0.4142	0.4460	0.017*	
N4	0.24150 (15)	0.51394 (5)	0.46181 (11)	0.01305 (18)	
C4A	0.21228 (17)	0.54833 (5)	0.32729 (13)	0.0128 (2)	
C5	0.19297 (18)	0.52421 (6)	0.18123 (13)	0.0145 (2)	
H5	0.2035	0.4799	0.1647	0.017*	
C6	0.15793 (18)	0.56688 (6)	0.06069 (13)	0.0151 (2)	
H6	0.1450	0.5522	−0.0405	0.018*	
C7	0.14192 (17)	0.63163 (6)	0.08972 (13)	0.0141 (2)	
N7	0.09229 (16)	0.67506 (5)	−0.04063 (12)	0.0179 (2)	
O1	0.03830 (18)	0.65210 (5)	−0.16990 (11)	0.0285 (2)	
O2	0.10321 (17)	0.73262 (5)	−0.01562 (12)	0.0264 (2)	
C8	0.16850 (18)	0.65707 (5)	0.23520 (13)	0.0147 (2)	
H8	0.1621	0.7016	0.2514	0.018*	
C8A	0.20485 (17)	0.61398 (6)	0.35558 (13)	0.0137 (2)	
S9	0.23601 (5)	0.631304 (14)	0.55005 (3)	0.01708 (8)	
C9A	0.25920 (18)	0.55010 (6)	0.59108 (13)	0.0146 (2)	
C10	0.31635 (17)	0.39985 (5)	0.77247 (13)	0.0136 (2)	
C11	0.37199 (18)	0.41148 (6)	0.92960 (14)	0.0156 (2)	
H11	0.3843	0.4540	0.9661	0.019*	
C12	0.4096 (2)	0.36113 (6)	1.03320 (14)	0.0191 (2)	
H12	0.4474	0.3695	1.1398	0.023*	
C13	0.3919 (2)	0.29863 (6)	0.98110 (15)	0.0203 (2)	
H13	0.4205	0.2644	1.0517	0.024*	
C14	0.3318 (2)	0.28656 (6)	0.82445 (15)	0.0202 (2)	
H14	0.3163	0.2440	0.7884	0.024*	
C15	0.29452 (19)	0.33662 (6)	0.72114 (14)	0.0169 (2)	
H15	0.2539	0.3280	0.6147	0.020*	

### Atomic displacement parameters (Å^2^)

**Table:**

	*U*^11^	*U*^22^	*U*^33^	*U*^12^	*U*^13^	*U*^23^
N1	0.0196 (5)	0.0129 (4)	0.0138 (4)	0.0008 (3)	0.0038 (4)	0.0005 (3)
C2	0.0125 (5)	0.0133 (5)	0.0148 (5)	0.0005 (4)	0.0037 (4)	0.0006 (4)
C3	0.0156 (5)	0.0124 (5)	0.0151 (5)	0.0005 (4)	0.0047 (4)	0.0003 (4)
N4	0.0148 (4)	0.0122 (4)	0.0120 (4)	0.0009 (3)	0.0033 (3)	0.0004 (3)
C4A	0.0120 (5)	0.0129 (5)	0.0137 (5)	−0.0003 (4)	0.0037 (4)	0.0012 (4)
C5	0.0160 (5)	0.0137 (5)	0.0142 (5)	0.0004 (4)	0.0049 (4)	−0.0005 (4)
C6	0.0159 (5)	0.0162 (5)	0.0134 (5)	−0.0002 (4)	0.0045 (4)	0.0001 (4)
C7	0.0140 (5)	0.0150 (5)	0.0136 (5)	0.0004 (4)	0.0043 (4)	0.0026 (4)
N7	0.0195 (5)	0.0187 (5)	0.0164 (5)	0.0027 (4)	0.0063 (4)	0.0035 (4)
O1	0.0448 (6)	0.0278 (5)	0.0129 (4)	0.0087 (5)	0.0076 (4)	0.0022 (4)
O2	0.0387 (6)	0.0155 (4)	0.0248 (5)	−0.0007 (4)	0.0076 (4)	0.0058 (4)
C8	0.0159 (5)	0.0131 (5)	0.0157 (5)	−0.0003 (4)	0.0053 (4)	0.0008 (4)
C8A	0.0146 (5)	0.0132 (5)	0.0131 (5)	−0.0002 (4)	0.0033 (4)	−0.0002 (4)
S9	0.02653 (16)	0.01152 (13)	0.01288 (13)	0.00037 (10)	0.00455 (11)	−0.00043 (9)
C9A	0.0173 (5)	0.0131 (5)	0.0132 (5)	0.0002 (4)	0.0036 (4)	−0.0010 (4)
C10	0.0127 (5)	0.0134 (5)	0.0152 (5)	0.0008 (4)	0.0044 (4)	0.0020 (4)
C11	0.0165 (5)	0.0160 (5)	0.0146 (5)	−0.0011 (4)	0.0044 (4)	0.0010 (4)
C12	0.0205 (6)	0.0218 (6)	0.0153 (5)	−0.0011 (4)	0.0050 (4)	0.0032 (4)
C13	0.0234 (6)	0.0180 (5)	0.0209 (6)	0.0008 (4)	0.0083 (5)	0.0068 (4)
C14	0.0264 (6)	0.0142 (5)	0.0217 (6)	0.0009 (4)	0.0090 (5)	0.0022 (4)
C15	0.0203 (5)	0.0144 (5)	0.0164 (5)	0.0016 (4)	0.0056 (4)	0.0009 (4)

### Geometric parameters (Å, º)

**Table:**

N1—C9A	1.3112 (15)	N7—O1	1.2323 (15)
N1—C2	1.4038 (15)	C8—C8A	1.3905 (16)
C2—C3	1.3795 (16)	C8—H8	0.9500
C2—C10	1.4666 (16)	C8A—S9	1.7590 (12)
C3—N4	1.3865 (15)	S9—C9A	1.7457 (12)
C3—H3	0.9500	C10—C11	1.3977 (16)
N4—C9A	1.3761 (15)	C10—C15	1.4034 (16)
N4—C4A	1.3873 (14)	C11—C12	1.3942 (17)
C4A—C5	1.3924 (16)	C11—H11	0.9500
C4A—C8A	1.4073 (16)	C12—C13	1.3909 (19)
C5—C6	1.3861 (16)	C12—H12	0.9500
C5—H5	0.9500	C13—C14	1.3957 (19)
C6—C7	1.3962 (17)	C13—H13	0.9500
C6—H6	0.9500	C14—C15	1.3880 (17)
C7—C8	1.3913 (16)	C14—H14	0.9500
C7—N7	1.4621 (15)	C15—H15	0.9500
N7—O2	1.2298 (15)		
			
C9A—N1—C2	103.89 (10)	C7—C8—H8	121.7
C3—C2—N1	111.14 (10)	C8—C8A—C4A	120.22 (11)
C3—C2—C10	128.20 (11)	C8—C8A—S9	127.08 (9)
N1—C2—C10	120.64 (10)	C4A—C8A—S9	112.66 (9)
C2—C3—N4	105.00 (10)	C9A—S9—C8A	89.54 (5)
C2—C3—H3	127.5	N1—C9A—N4	113.29 (10)
N4—C3—H3	127.5	N1—C9A—S9	134.63 (9)
C9A—N4—C3	106.68 (10)	N4—C9A—S9	112.07 (8)
C9A—N4—C4A	114.97 (10)	C11—C10—C15	118.77 (11)
C3—N4—C4A	138.35 (10)	C11—C10—C2	120.14 (10)
N4—C4A—C5	127.12 (10)	C15—C10—C2	121.09 (11)
N4—C4A—C8A	110.77 (10)	C12—C11—C10	120.52 (11)
C5—C4A—C8A	122.12 (10)	C12—C11—H11	119.7
C6—C5—C4A	117.98 (11)	C10—C11—H11	119.7
C6—C5—H5	121.0	C13—C12—C11	120.25 (12)
C4A—C5—H5	121.0	C13—C12—H12	119.9
C5—C6—C7	119.22 (11)	C11—C12—H12	119.9
C5—C6—H6	120.4	C12—C13—C14	119.61 (12)
C7—C6—H6	120.4	C12—C13—H13	120.2
C8—C7—C6	123.81 (11)	C14—C13—H13	120.2
C8—C7—N7	118.19 (10)	C15—C14—C13	120.21 (12)
C6—C7—N7	118.00 (10)	C15—C14—H14	119.9
O2—N7—O1	123.34 (11)	C13—C14—H14	119.9
O2—N7—C7	118.37 (11)	C14—C15—C10	120.61 (11)
O1—N7—C7	118.28 (11)	C14—C15—H15	119.7
C8A—C8—C7	116.54 (11)	C10—C15—H15	119.7
C8A—C8—H8	121.7		
			
C9A—N1—C2—C3	−0.18 (13)	N4—C4A—C8A—S9	0.33 (12)
C9A—N1—C2—C10	178.54 (10)	C5—C4A—C8A—S9	−179.35 (9)
N1—C2—C3—N4	0.39 (13)	C8—C8A—S9—C9A	177.73 (11)
C10—C2—C3—N4	−178.21 (11)	C4A—C8A—S9—C9A	0.12 (9)
C2—C3—N4—C9A	−0.43 (12)	C2—N1—C9A—N4	−0.11 (14)
C2—C3—N4—C4A	−179.63 (13)	C2—N1—C9A—S9	178.45 (11)
C9A—N4—C4A—C5	178.88 (11)	C3—N4—C9A—N1	0.36 (14)
C3—N4—C4A—C5	−2.0 (2)	C4A—N4—C9A—N1	179.77 (10)
C9A—N4—C4A—C8A	−0.78 (14)	C3—N4—C9A—S9	−178.54 (8)
C3—N4—C4A—C8A	178.38 (13)	C4A—N4—C9A—S9	0.88 (13)
N4—C4A—C5—C6	177.96 (11)	C8A—S9—C9A—N1	−179.12 (13)
C8A—C4A—C5—C6	−2.41 (17)	C8A—S9—C9A—N4	−0.55 (9)
C4A—C5—C6—C7	−0.48 (17)	C3—C2—C10—C11	170.54 (12)
C5—C6—C7—C8	3.11 (18)	N1—C2—C10—C11	−7.93 (17)
C5—C6—C7—N7	−176.24 (10)	C3—C2—C10—C15	−8.38 (18)
C8—C7—N7—O2	9.70 (17)	N1—C2—C10—C15	173.14 (11)
C6—C7—N7—O2	−170.91 (11)	C15—C10—C11—C12	1.45 (17)
C8—C7—N7—O1	−169.09 (11)	C2—C10—C11—C12	−177.50 (11)
C6—C7—N7—O1	10.30 (16)	C10—C11—C12—C13	−0.06 (19)
C6—C7—C8—C8A	−2.66 (17)	C11—C12—C13—C14	−1.4 (2)
N7—C7—C8—C8A	176.69 (10)	C12—C13—C14—C15	1.5 (2)
C7—C8—C8A—C4A	−0.31 (17)	C13—C14—C15—C10	−0.10 (19)
C7—C8—C8A—S9	−177.77 (9)	C11—C10—C15—C14	−1.37 (18)
N4—C4A—C8A—C8	−177.46 (10)	C2—C10—C15—C14	177.57 (11)
C5—C4A—C8A—C8	2.86 (17)		
